# Metabolomics-Based Analysis Linking Oxidative Stress-Related Branched-Chain Amino Acid (BCAA) Pathway with Atopic Indices to Childhood Allergies

**DOI:** 10.3390/antiox15060720

**Published:** 2026-06-05

**Authors:** Jin-Ling Ku, Kuan-Wen Su, Meng-Han Chiang, Chieh-Ni Kuo, Kuo-Wei Yeh, Jing-Long Huang, Chih-Yung Chiu

**Affiliations:** 1 Department of Medical Education, Chang Gung Memorial Hospital at Kaohsiung, Kaohsiung 833401, Taiwan; elsiejlk1111@gmail.com; 2 Department of Pediatrics, Chang Gung Memorial Hospital at Linkou, Chang Gung University, Taoyuan 333423, Taiwan; b87401102@cgmh.org.tw (K.-W.S.); kuochiehni@gmail.com (C.-N.K.); kwyeh@adm.cgmh.org.tw (K.-W.Y.); hjlong0182@gmail.com (J.-L.H.); 3 Multi-Omics Core Laboratory, Chang Gung Memorial Hospital at Linkou, Taoyuan 33302, Taiwan; 0914.neo@gmail.com

**Keywords:** metabolomics, oxidative stress, childhood allergies, BCAA metabolism, threonine, immunoglobulin E

## Abstract

Allergic diseases are complex conditions in which oxidative stress contributes to pathogenesis, yet the metabolic mechanisms linking oxidative stress to immunoglobulin E (IgE)-mediated responses remain unclear. This study analyzed 124 children at an 8-year follow-up, identifying those with eczema, rhinitis, and asthma. Oxidative stress markers and ^1^H-nuclear magnetic resonance (NMR) blood metabolomic profiles were assessed to determine associations between metabolic pathways and atopic indices. Results showed that glutathione peroxidase (GPx) activity was significantly lower in seafood-sensitized children, while FeNO and mite-specific IgE were elevated in children with rhinitis (*p* < 0.01). Fractional exhaled nitric oxide (FeNO) correlated positively with allergen-specific IgE and negatively with 8-hydroxy-2′-deoxyguanosine (8-OHdG) (*p* < 0.01) and rhinitis-related methionine. Furthermore, seafood-specific IgE showed negative correlations with glucose and threonine (*p* < 0.01). Among 22 metabolites linked to atopy, threonine correlated positively with GPx (*p* < 0.01), while serine and mannose were associated with total antioxidant capacity (TAC). Pathway analysis revealed that branched-chain amino acid (BCAA) and glycine-serine-threonine metabolism intersected significantly with oxidative stress and atopic indices. In conclusion, a metabolomics-based approach highlights that oxidative stress-related BCAA and threonine pathways are central to the metabolic signature of childhood allergies, providing potential targets for future therapeutic interventions.

## 1. Introduction

Allergic diseases are immune-mediated disorders with multifactorial etiologies [1,2]. Clinically, allergen exposure triggers an IgE-mediated response involving various inflammatory cells, including mast cells and eosinophils [3,4]. Oxidative stress results from excessive reactive oxygen species (ROS) generation relative to antioxidant defenses and has been increasingly implicated in the pathogenesis of asthma, allergic rhinitis, and atopic dermatitis [5,6,7,8]. Foundational studies from the early 2000s established that oxidative stress in the airways is not merely a byproduct but also a primary mediator of allergic inflammation [9,10,11]. Specifically, allergic reactions induce a ‘respiratory burst’ in activated inflammatory cells through nicotinamide adenine dinucleotide phosphate (NADPH) oxidase activation, releasing ROS that subsequently stimulate nuclear factor-κB-mediated pro-inflammatory signaling [9,12]. When these oxidative insults overwhelm endogenous antioxidant systems, such as superoxide dismutase (SOD) and glutathione (GSH), the resulting redox imbalance exacerbates mucosal inflammation and airway hyperresponsiveness [10,11,13]. Despite these insights, the mechanisms and the specific involvement of oxidative stress in IgE-sensitized allergic diseases are complex and remain insufficiently characterized.

Metabolomic profiling of biofluid samples is widely applied in allergic disease research to identify biomarkers for early diagnosis, potential causal pathways, and therapeutic strategies [8]. This approach captures changes in small molecules, offering comprehensive insights into biological function and regulation [13]. NMR spectroscopy is commonly used because of its high reproducibility and comprehensive molecular profiling capacity [14]. Recent pediatric thoracic research has further highlighted the utility of NMR–and liquid chromatography–mass spectrometry (LC-MS)-based metabolomics for identifying disease-related metabolic signatures and advancing precision medicine approaches in pediatric respiratory and critical illnesses [15]. Blood metabolic profiles appropriately reflect systemic metabolic states and are therefore suitable for assessing overall health [16].

This study aimed to investigate the biological significance of oxidative stress in childhood allergies by performing metabolomic analysis using ^1^H-NMR spectroscopy in a birth cohort. By integrating metabolomic analysis with functional pathway assessment, we sought to elucidate the molecular mechanisms linking oxidative stress, atopic indices, and allergic diseases, while exploring potential strategies for disease management. Our findings demonstrate that specific metabolic signatures, particularly within the BCAA and glycine-serine-threonine pathways, are significantly associated with antioxidant capacity and allergen-specific sensitization. These results highlight a complex interplay in which oxidative stress-related metabolic alterations contribute to the pathogenesis of childhood allergies and may serve as potential biomarkers for clinical monitoring.

## 2. Materials and Methods

### 2.1. Study Population

This study included children delivered at Keelung Chang Gung Memorial Hospital (CGMH) who were enrolled in a specific birth cohort and completed an 8-year follow-up [17]. For each participant at the 8-year follow-up, two biological specimens were collected from each participant: one fasting peripheral venous blood sample and one midstream urine sample. To maintain the sample integrity and chain of custody, all specimens were labeled with unique study identification numbers and immediately transported on ice to the laboratory for processing. Comprehensive demographic data, including passive smoking exposure, household income, and family history of atopy were systematically collected and analyzed. Atopic diseases investigated—eczema, allergic rhinitis, and asthma—were diagnosed by a pediatric pulmonologist based on the criteria used in our previous study [18]. To minimize confounding, subjects with overlapping atopic diseases were excluded. While these conditions often coexist in early childhood, they typically manifest as a single predominant disease by age 8; therefore, we focused on the primary diagnosis at the 8-year follow-up. Ethical approval for this study was obtained from the Ethics Committee of Chang Gung Memorial Hospital (No. 104-8925C, approval date: 28 April 2016). All procedures adhered to the relevant guidelines and regulations, with written informed consent provided by a parent or legal guardian.

### 2.2. Assessment of Serum Total and Allergen-Specific IgE

Serum IgE levels were quantified using ImmunoCAP (Phadia, Uppsala, Sweden) according to the manufacturer’s protocols. Serum allergen-specific IgE levels were measured using a commercial assay (ImmunoCAP Phadiatop Infant, Phadia) targeting food and inhalant allergens, including crab, shrimp, *Dermatophagoides farinae*, and *Dermatophagoides pteronyssinus* [19]. The measurable range for allergen-specific IgE was 0.10 to 100 kU/L, and values exceeding 100 kU/L were assigned a value of 100 kU/L. An allergen-specific IgE concentration exceeding 0.35 kU/L was considered indicative of sensitization [20].

### 2.3. Measurement of Oxidative Stress Markers

Blood samples were drawn into ethylenediaminetetraacetic acid (EDTA) tubes, and plasma was separated within 2 h by centrifugation at 3000× *g* for 10 min at 4 °C. Plasma and urine samples were then divided into aliquots and maintained at −80 °C until analysis for oxidative stress markers and NMR spectroscopy.

Plasma GPx activity were measured using a Randox Laboratories LTDA reagent kit and the Thermo Indiko chemical analyzer (ABX, Montpellier, France) by monitoring NADPH oxidation to NADP^+^ at a wavelength of 340 nm.

Plasma myeloperoxidase (MPO) concentrations were quantified using Bioxytech MPO enzyme-linked immunosorbent assay (ELISA) kit (Oxis International Inc., Portland, OR, USA) following the manufacturer’s protocol and using 4-nitrophenylphosphate as a substrate. MPO levels below the detection limit were recorded as 48.4 ng/mL.

Urinary 8-OHdG levels were determined with an 8-OHdG ELISA kit (Japan Institute for the Control of Aging, Fukuroi, Japan) in accordance with the manufacturer guidelines.

TAC in plasma was assessed using a ferric reducing antioxidant power (FRAP) assay with a Thermo Indiko analyzer (ABX, Montpellier, France), based on the reduction in ferric (Fe^3+^) ions.

### 2.4. Measurement of FeNO

Expiratory FeNO was assessed with the NIOX MINO device (Aerocrine AB, Solna, Sweden) in accordance with the recommendations of European Respiratory Society (ERS) and the American Thoracic Society (ATS) [21]. FeNO assessment was performed prior to spirometry and bronchodilator reversibility testing. Levels below 20 parts per billion (ppb) were classified as normal, whereas levels ≥ 20 ppb indicated eosinophilic inflammation [21].

### 2.5. ^1^H–NMR Spectroscopic Analysis

As described previously [22], plasma samples for spectral acquisition were prepared by mixing 500 μL of plasma with phosphate buffer in deuterium water containing 0.08% 3-(trimethylsilyl)-propionic-2,2,3,3-d4 acid sodium salt (TSP). The mixture was vortexed for 20 s and centrifuged at 12,000× *g* at 4 °C for 30 min, after which 600 μL of the supernatant was transferred into a 5 mm NMR tube.

^1^H-NMR spectroscopic analysis was conducted at the Chang Gung Healthy Aging Research Center in Taiwan using a Bruker Avance 600 MHz spectrometer (Bruker-Biospin GmbH, Karlsruhe, Germany). A total of 64 scans were recorded into 64K data points over a spectral width of 10,000 Hz (10 parts per million, ppm). Spectral processing involved zero-filled Fourier transformation with a 0.3 Hz line broadening factor, manual phasing, baseline correction, and chemical-shift referencing to TSP (δ 0.0 ppm) using TopSpin 3.2 software (Bruker BioSpin, Rheinstetten, Germany).

### 2.6. NMR Spectral Processing and Data Analysis

Raw ^1^H-NMR spectra were analyzed using open-source software NMRProcFlow v1.4, which was applied for spectral processing, ppm calibration, baseline correction, alignment, spectral bucketing, and normalization [23]. Spectral misalignment was corrected using a least-squares algorithm in combination with parametric time warping. Spectral bucketing was performed with an intelligent bucketing approach using variable bin sizes [24]. Metabolites assignment was performed using Chenomx NMR Suite 11 software (Chenomx Inc., Edmonton, AB, Canada). To account for concentration variability, urine spectra were normalized against the creatinine peak at δ 3.045 ppm.

After generalized log transformation (glog), the normalized ^1^H-NMR spectra were uploaded to MetaboAnalyst 6.0. Partial least squares-discriminant analysis (PLS-DA) was then applied to identify metabolites capable of differentiating between groups. Mean-centering and Pareto scaling were applied to the spectral variables, followed by 10-fold cross-validation to assess model robustness using R^2^ and Q^2^ values. Metabolites meeting either criterion—a variable importance in projection (VIP) score ≥ 1.0 or a *p*-value < 0.05—were retained for further analysis. The Kyoto Encyclopedia of Genes and Genomes (KEGG) database was used to examine the related functional metabolic pathways.

### 2.7. Statistical Analyses

Demographic information was collected through questionnaires to describe population characteristics and the proportion of physician-diagnosed atopic diseases. Continuous variables were compared using Student’s *t*-test or the Mann–Whitney U test. When more than two groups were compared, analysis of variance (ANOVA) with Tukey’s post hoc test or the Kruskal–Wallis test with Bonferroni-adjusted pairwise comparisons was employed. The chi-square test was applied to compare categorical variables, while Spearman’s correlation analysis was performed to examine relationships among oxidative stress markers, allergen-specific IgE levels, and metabolites. Random Forest classifier models with Boruta feature selection and 20-fold stratified cross-validation were used to determine the most influential features. IBM SPSS Statistics for Windows, Version 25.0 (IBM, Armonk, NY, USA) was used for all statistical analyses. Figures were generated with GraphPad Prism Version 8.01 (GraphPad Software Inc., San Diego, CA, USA), whereas Venn diagrams were produced using Venny 2.1.0. Statistical significance was defined as a two-sided *p*-value < 0.05.

## 3. Results

### 3.1. Population Characteristics

A total of 258 subjects were initially recruited into a birth cohort, of whom 155 (60.1%) completed an 8-year clinical follow-up and were enrolled in this study. Among them, 23 children diagnosed with combinations of eczema, asthma, or rhinitis were excluded. The final cohort consisted of 50 healthy controls and 7, 58, and 9 children with eczema, allergic rhinitis, and asthma, respectively. Among the 50 healthy controls, 20 (40%) and 7 (14%) children were sensitized to mites and seafood, respectively, without clinical symptoms of atopy. Table 1 shows the differences in baseline characteristics among the groups. Children with atopic diseases had significantly higher body weight and body mass index (BMI) than controls (*p* < 0.05). Furthermore, seafood-specific IgE levels were significantly higher only in children with rhinitis (*p* < 0.05), whereas mite-specific IgE levels (*p* < 0.001) and total serum IgE levels (*p* < 0.001) were significantly higher in atopic groups. No statistically significant differences were identified in age, sex, maternal atopy, passive smoking exposure, household income, or oxidative stress markers.

### 3.2. Associations Among Oxidative Stress Markers, Allergen-Specific IgE, FeNO Level, Allergen Sensitization, and Atopic Diseases

Children with seafood sensitization exhibited significantly lower GPx activity (*p* < 0.05) than those without seafood sensitization (Figure S1A). No significant associations were found between other oxidative stress markers and atopic indices. Figure S2 presents variations in total serum IgE, allergen-specific IgE, and FeNO levels across children with different atopic diseases. Compared with healthy controls, total IgE and mite-specific IgE levels were significantly higher in children with rhinitis and asthma (*p* < 0.01, Figure S2A–C). Crab-specific IgE levels (Figure S2E, *p* < 0.05) and FeNO levels (Figure S2F, *p* < 0.001) were significantly higher in children with rhinitis.

### 3.3. Identification of Blood Metabolites for the Different Atopic Diseases and Atopic Indices

In the total study group (*n* = 124), plasma ^1^H-NMR data analysis identified 1000 buckets across the NMR spectrum, of which 93 buckets linked to 39 known metabolites using Chenomx NMR Suite. Metabolites that differentiated the study groups were identified using PLS-DA, and the corresponding analytical results and score plots are provided in Table S1 and Figure S3, respectively. Metabolites selected for atopic diseases using a cutoff of VIP score > 1.00, false discovery rate (FDR)-adjusted *p*-values, and fold change are shown in Table S2. Pyruvic acid, creatinine, and methionine were significantly differentially expressed in children with eczema and rhinitis (*p* < 0.05). All children were stratified according to the presence or absence of different atopic indices, and metabolites associated with each atopic index were subsequently identified and compared. Table 2 shows 22 metabolites significantly that differed significantly according to atopic indices (FDR-adjusted *p* < 0.05). Sixteen metabolites were significantly associated with total IgE sensitization (*p* < 0.05). Furthermore, acetic acid, threonine, and glucose were significantly associated with seafood sensitization, whereas ethanol, dimethyl sulfone, histidine, glycine, asparagine, and creatinine were associated with mite sensitization (*p* < 0.05). Moreover, ethanol, isoleucine, and isovaleric acid were strongly associated with higher FeNO levels (≥20 ppb) (*p* < 0.01).

### 3.4. Metabolic Pathway Related to Different Atopic Indices

Table S3 presents metabolic functional pathways related to atopic indices. IgE sensitization (>100 kU/L) was significantly associated with glutamic acid-related metabolic pathways, including alanine, aspartate, and glutamate metabolism, glyoxylate and dicarboxylate metabolism, and butanoate metabolism (*p* < 0.001). Seafood sensitization was significantly associated with acetic acid–and glucose-related glycolysis/gluconeogenesis metabolism (*p* < 0.001), whereas mite sensitization was significantly associated with histidine metabolism (*p* < 0.05). Furthermore, higher FeNO levels (≥20 ppb) were strongly correlated with valine, leucine, and isoleucine biosynthesis and degradation pathways (*p* < 0.001).

### 3.5. Metabolites Associated with Atopic Indices Related to Allergen-Specific IgE and Oxidative Stress Levels

**Figure 1:**
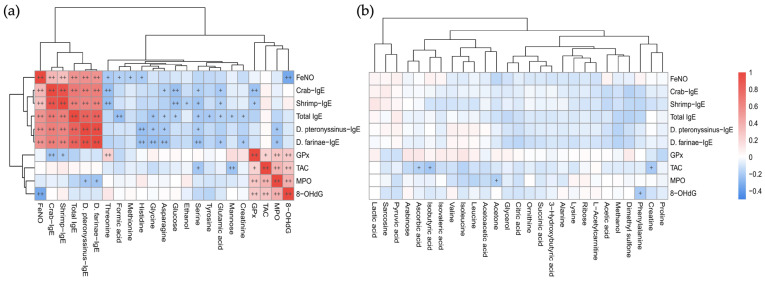
Heatmap of Spearman’s correlations of (**a**) atopic indices related metabolites with allergen-specific IgE levels and oxidative stress levels; (**b**) metabolites unrelated to allergen-specific IgE levels and oxidative stress levels. Metabolites are separated into (**a**,**b**) based on their statistical significance to prioritize the presentation of key findings and ensure figure legibility. Analysis was performed on all study participants (*n* = 124) to capture metabolic transitions. Color intensity represents the magnitude of correlation. + symbol means a *p*-value < 0.05; ++ symbol means a *p*-value < 0.01. FeNO, fractional exhaled nitric oxide; IgE, immunoglobulin E; *D. pteronyssinus*, *Dermatophagoides pteronyssinus*; *D. farinae*, *Dermatophagoides farinae*; GPx, glutathione peroxidase; TAC, total anti-oxidant capacity; MPO, myeloperoxidase; 8-OHdG, 8-hydroxy-2′-deoxyguanosine.

To ensure figure legibility and prioritize clinically relevant findings, the identified metabolites were stratified into two groups for correlation analysis based on their statistical significance. Among all 124 subjects, correlations among atopic index-related metabolites with allergen-specific IgE levels and oxidative stress levels are shown in Figure 1a. Correlation analyses included all subjects to capture metabolic transitions from asymptomatic sensitization to clinical atopic diseases. Positive correlations were observed atopic indices and among oxidative stress markers themselves (*p* < 0.01). FeNO levels were positively correlated with allergen-specific IgE and negatively with 8-OHdG levels (*p* < 0.01) and rhinitis-related methionine (*p* < 0.05). Additionally, crab- and shrimp-specific IgE levels were significantly negatively correlated with glucose and threonine (*p* < 0.01) and significantly positively correlated with GPx (*p* < 0.05). Mite-specific IgE levels were negatively correlated with histidine (*p* < 0.01), glycine, asparagine, serine (*p* < 0.05), and MPO levels (*p* < 0.05). Furthermore, threonine was positively correlated with GPx (*p* < 0.01), whereas serine and mannose were associated with TAC (*p* < 0.05). Figure 1b presents the correlations of metabolites unrelated to allergen-specific IgE levels, showing a few metabolites weakly correlated with oxidative stress markers but had no identified functional pathways, suggesting a lesser association of oxidative stress with non-IgE pathways.

### 3.6. Metabolic Pathway and Function Analysis of Oxidative Stress-Related Atopic Indices Correlated Metabolites

**Figure 2:**
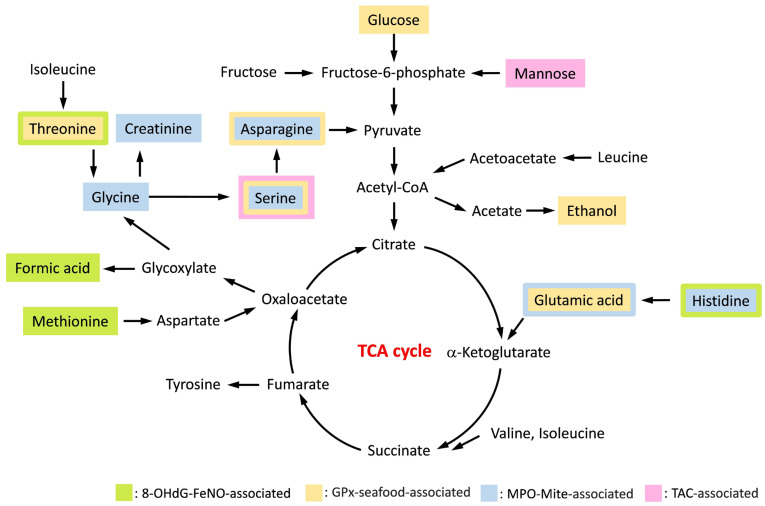
Schematic overview of metabolic pathways of oxidative stress related different atopic indices correlated metabolites. TCA cycle, tricarboxylic acid cycle.

Table 3 illustrates the functional pathways of metabolites correlated with different atopic indices related to oxidative stress. Amino acid metabolism, including valine, leucine, and isoleucine biosynthesis, as well as histidine metabolism, showed significant associations with 8-OHdG related FeNO correlated metabolites (*p* < 0.05). GPx related seafood correlated metabolites showed significant associations with threonine related glycine, serine and threonine metabolism (*p* < 0.01), as well as valine, leucine and isoleucine biosynthesis (*p* < 0.05). Meanwhile, MPO-related mite-correlated metabolites were significantly associated with glyoxylate and dicarboxylate metabolism and glycine, serine and threonine metabolism (*p* < 0.01). Figure 2 presents comprehensive metabolic functional pathways of oxidative stress-related atopic indices correlated metabolites, illustrating the proposed molecular mechanisms linking oxidative stress to allergies.

## 4. Discussion

Urinary 8-OHdG, an oxidative stress marker reflecting oxidative DNA damage, has been associated with childhood atopic diseases [25]. In the present study, 8-OHdG was also found to be negatively correlated with FeNO [26], which reflects ongoing airway inflammation and is elevated in pediatric asthma [27]. Additionally, asthmatic patients with high FeNO levels have been reported to exhibit elevated plasma BCAA [28], which are strongly correlated with pro-inflammatory and oxidative status [29]. Notably, this study identified a significant association between valine, leucine and isoleucine (BCAA) biosynthesis related to FeNO associated with 8-OHdG. These findings suggest BCAA metabolism associated with FeNO-related airway inflammation could particularly explain the strong association between urinary 8-OHdG and allergic diseases.

FeNO should be interpreted as a marker reflecting both allergic and non-allergic components of type 2 airway immunity. In allergic type 2 inflammation, allergen sensitization promotes Th2-mediated responses, with interleukin (IL)-4 contributing to IgE class switching and IL-13 inducing nitric oxide production in airway epithelial cells [30,31]. This is consistent with our finding that FeNO was positively correlated with allergen-specific IgE and elevated in children with rhinitis [32]. However, FeNO may also reflect airway-localized type 2 inflammation rather than systemic type 2 activation alone. A recent study of individuals naturally exposed to helminth parasites showed that healthy children from rural areas showed the lowest FeNO levels, despite having the highest total IgE levels, plasma IL-5 levels, and eosinophil counts. This finding suggests that, during type 2 responses, FeNO may be influenced by lung-specific regulatory mechanisms controlling inducible nitric oxide synthase (iNOS) expression [33]. Therefore, the FeNO-associated BCAA metabolic signature observed in this study may reflect a shared downstream IL-13/iNOS-related pathway linking allergic sensitization, airway-localized type 2 inflammation, and oxidative stress. Because cytokines and cellular type 2 markers were not directly measured, future studies should include IL-4/IL-5/IL-13 levels, eosinophil counts, epithelial alarmins, and type 2 innate lymphoid cell (ILC2)-related biomarkers to clarify these mechanisms [34,35].

GPx, a crucial selenoprotein, reduces hydrogen peroxide and lipid hydroperoxides, thereby protecting mucosal epithelial cells from oxidative stress [36]. Threonine, which is essential for protein synthesis and mucosal barrier maintenance [37], showed a significant positive correlation with GPx in this study. Seafood is an important dietary source of selenium [38], and selenium availability is closely linked to GPx activity [36]. The inverse association between threonine and seafood-specific IgE, together with the positive correlation between threonine and GPx, suggests a potential interaction between amino acid metabolism and selenium-dependent antioxidant defense in sensitized children. These findings raise the possibility that modulation of threonine metabolism, including dietary supplementation, may represent a potential strategy for enhancing antioxidant protection in allergic diseases.

Furthermore, our study identified acetic acid as a metabolite significantly associated with seafood sensitization, whereas no significant correlations were found with other atopic indices or oxidative stress markers, such as GPx, MPO, and urinary 8-OHdG. This lack of association suggests that acetic acid may function as a specific metabolic signature for diet-related sensitization rather than as a general indicator of redox imbalance in childhood allergies. Acetic acid is a major short-chain fatty acid (SCFA) generated predominantly by the gut microbial through the fermentation of dietary fiber. SCFAs are well known for their immunomodulatory properties, particularly in promoting the differentiation of regulatory T cells and maintaining epithelial barrier integrity [39,40]. Although previous research has emphasized the protective role of acetate in general airway inflammation [41], our findings in this 8-year-old cohort suggest a potential diet-microbiome-metabolite axis specific to food-related allergies. This independent relationship highlights the complexity of the metabolome, in which certain metabolites reflect allergen-specific pathways that are distinct from mechanisms of oxidative damage, underscoring the importance of considering allergen-specific metabolic signatures in pediatric populations.

MPO, a heme-containing peroxidase present in neutrophil extracellular traps (NETs) [42], generates hypochlorous acid (HOCl), which has antibacterial and anti-inflammatory effects [43]. Iron status is linked to allergic and respiratory diseases [44,45], and disturbances in iron-related oxidative pathways may contribute to inflammatory responses in mite-sensitized individuals [46]. In this study, several amino acids, including histidine, glycine, asparagine, and serine, were strongly correlated with mite-specific IgE levels but not with MPO, indicating the presence of MPO-independent metabolic pathways in mite-induced allergic reactions. Notably, the negative correlation between MPO and mite-specific IgE levels in our cohort suggests that MPO may be involved in regulating oxidative stress-related allergic inflammation among mite-sensitized children.

TAC represents the collective ability of antioxidants to neutralize ROS and prevent oxidative damage [47], with lower levels reported in patients with asthma [48,49]. In the present study, TAC was assessed using the ferric reducing antioxidant power assay, which reflects the capacity of plasma antioxidants to convert ferric (Fe^3+^) ions into ferrous (Fe^2+^) ions. Recent reviews have highlighted that iron status may influence atopic and respiratory diseases through its effects on immune regulation, type 2 inflammation, IgE production, and allergen-associated iron-binding mechanisms [50,51]. Mannose, known for its immune-modulatory properties, can reduce airway inflammation [52,53]. In this study, although TAC was not significantly associated with IgE levels, mannose showed a strong positive correlation with TAC, suggesting that mannose-related metabolic changes may be linked to antioxidant capacity in children with oxidative stress-related allergic diseases. These findings may provide a basis for future studies investigating whether mannose-related metabolic modulation contributes to antioxidant defense or airway inflammation control in pediatric allergic diseases. However, because direct iron biomarkers were not measured, the relationship among iron status, TAC, and allergic inflammation should be interpreted as hypothesis-generating.

Methionine is an essential amino acid trans-sulfurated to GSH [54], a powerful antioxidant that protects the airways from oxidative stress and inflammation [55]. Reduced GSH levels have been observed in children with allergic rhinitis [56]. Meanwhile, nitric oxide (NO) inhibits methionine synthesis [57,58]. In this study, methionine was associated with allergic rhinitis and negatively correlated with FeNO levels, supporting a potential association between methionine metabolism and allergic diseases. This association may be influenced by oxidative stress-related airway inflammation.

This study is limited primarily by its relatively small sample size, particularly among children with eczema (*n* = 7) and asthma (*n* = 9). The limited number of subjects in these specific groups posed substantial challenges for performing robust subgroup analyses and may have reduced the ability to identify statistically significant metabolic variations unique to these atopic phenotypes. Although the participants were derived from a birth cohort with follow-up through 8 years of age, the laboratory measurements and metabolomic analyses were performed at a single time point; therefore, the findings should be interpreted as cross-sectional associations rather than longitudinal changes. In addition, the present study did not include direct functional validation, such as in vitro or in vivo experiments, to confirm the causal roles of the identified metabolic pathways. Therefore, the associations involving BCAA metabolism, the GPx-threonine relationship, and other oxidative stress-related metabolites should be regarded as hypothesis-generating rather than mechanistically conclusive. Consequently, although the current results provide valuable preliminary insights, their interpretation should remain cautious until they are confirmed in larger cohorts. Additionally, ^1^H-NMR spectroscopy has limited sensitivity for detecting metabolites below 100 nmol/L using ^1^H-NMR spectroscopy. However, ^1^H-NMR spectroscopy provides good reproducibility and high-throughput molecular identification. The age-matched design also minimized variability in metabolic profiles. This study is strengthened by its birth cohort design with long-term follow-up and rigorous diagnostic evaluation of atopic diseases. In addition, the combined assessment of oxidative stress biomarkers and atopic indices enhances the validity and clinical relevance of the findings.

## 5. Conclusions

A metabolomics-based approach can help elucidate the complex interactions between oxidative stress and childhood allergies. Urinary 8-OHdG is strongly associated with FeNO-related airway inflammation, and BCAA metabolism may contribute to this association. GPx was significantly correlated with threonine, which is strongly associated with seafood-specific IgE levels in children with allergies. A strong correlation between MPO and mite-specific IgE levels suggests a potential role of MPO in modulating allergic inflammation in mite-sensitized children. The strong correlation between TAC and mannose suggests that TAC may be involved in airway inflammation. However, further functional studies are needed to investigate these associations in greater depth.

## Figures and Tables

**Table 1:** Epidemiological characteristics of the 124 children included in this study.

Characteristics	Controls	Eczema	Rhinitis	Asthma	*p* -Value
	(*n* = 50)	(*n* = 7)	(*n* = 58)	(*n* = 9)	
Age (yr)	7.9 ± 0.5	7.9 ± 0.6	8.0 ± 0.5	7.8 ± 0.8	0.719
Sex, male	24 (48.0%)	4 (57.1%)	36 (58.6%)	7 (77.8%)	0.368
Body height (percentile)	44.9 ± 4.5	57.0 ± 15.0	56.1 ± 3.5	59.5 ± 8.5	0.192
Body weight (percentile)	41.9 ± 4.6	63.5 ± 15.1	55.2 ± 4.0	74.4 ± 9.1 *	**0.014**
Body mass index (percentile)	41.2 ± 4.7	58.0 ± 15.3	50.7 ± 4.3	73.3 ± 9.8 *	**0.035**
Maternal atopy	29 (38.0%)	2 (28.6%)	28 (48.3%)	4 (44.4%)	0.631
Passive smoking	16 (32.0%)	4 (57.1%)	27 (46.6%)	4 (44.4%)	0.347
Household income					
Low, ≤500,000 NTD	18 (36.7%)	1 (14.3%)	21 (36.8%)	2 (28.6%)	
Medium, 500,000–1,000,000 NTD	23 (46.9%)	4 (57.1%)	27 (47.4%)	4 (57.1%)	
High, >1,000,000 NTD	8 (16.3%)	2 (28.6%)	9 (15.8%)	1 (14.3%)	
Allergen-specific IgE, kU/L					
Crab	0.8 ± 3.6	1.3 ± 2.5	0.7 ± 2.4	0.4 ± 0.8 *	**0.009**
Shrimp	1.0 ± 4.0	1.6 ± 3.5	0.8 ± 2.8	0.5 ± 1.0 *	**0.021**
*D. pteronyssinus*	9.3 ± 23.4	64.1 ± 38.4 *	35.1 ± 37.0 *	55.1 ± 45.8 *	**<0.001**
*D. farinae*	6.1 ± 16.9	46.3 ± 34.9 *	27.5 ± 32.7 *	44.7 ± 43.1 *	**<0.001**
Total IgE, kU/L	168.0 ± 351.6	613.2 ± 557.8 *	499.0 ± 842.1 *	592.3 ± 682.4 *	**<0.001**
Oxidative stress markers					
GPx, U/g	38.7 ± 15.5	39.0 ± 9.6	38.2 ± 10.5	36.9 ± 5.0	0.942
MPO, ng/mL	65.3 ± 35.3	57.3 ± 27.6	67.0 ± 46.9	48.4 ± 0.0	0.330
8-OHdG, ng/mg	67.3 ± 21.0	63.8 ± 17.5	61.9 ± 16.2	72.7 ± 18.3	0.399
TAC, umol/L	426.4 ± 69.6	441.3 ± 45.7	433.2 ± 64.5	456.8 ± 70.8	0.762

Data shown are mean ± SD or number (%) of patients as appropriate. yr, year; NTD, New Taiwan Dollar; IgE, immunoglobulin E; *D. pteronyssinus*, *Dermatophagoides pteronyssinus*; *D. farinae*, *Dermatophagoides farinae*; GPx, glutathione peroxidase; MPO, myeloperoxidase; 8-OHdG, 8-hydroxy-2′-deoxyguanosine; TAC, total anti-oxidant capacity. *p*-values were calculated using ANOVA with Tukey’s post hoc test or the Kruskal–Wallis test with Bonferroni-adjusted pairwise comparisons. * *p* < 0.05 compared to the healthy control group. All *p*-values < 0.05, which is in bold, are significant.

**Table 2:** VIP score and fold changes of metabolites showing significant differential expression according to atopic indices in the overall study population of 124 participants.

Metabolites	Chemical Shift, ppm	Seafood (+/−)			Mite (+/−)			Total IgE > 100 kU/L (+/−)			FeNO ≥ 20 ppb (+/−)		
		VIP Score *	Fold Change †	*p* ‡	VIP Score	Fold Change	*p*	VIP Score	Fold Change	*p*	VIP Score	Fold Change	*p*
Acetic acid	1.908–1.914 (s)	2.02	0.75	**0.026**	1.36	0.89	0.210	0.93	0.90	0.350	0.30	1.03	0.956
Threonine	4.232–4.237 (m)	1.66	0.88	**0.036**	0.60	0.96	0.395	1.14	0.90	**0.040**	1.26	1.03	0.932
Glucose	4.622–4.656 (d)	1.24	0.93	**0.044**	1.00	0.95	0.171	0.94	0.94	**0.037**	0.87	0.87	0.102
Ethanol	1.148–1.181 (t)	0.50	0.91	0.498	2.35	0.79	**0.007**	0.63	0.91	0.338	1.93	0.72	**<0.001**
Dimethyl sulfone	3.140–3.151 (s)	0.67	0.98	0.827	1.64	0.92	**0.015**	1.04	0.93	**0.022**	0.84	0.93	0.759
Histidine	7.759–7.800 (d)	1.27	0.98	0.884	1.46	0.92	**0.018**	0.98	0.93	**0.048**	1.01	0.96	0.424
Glycine	3.548–3.562 (s)	0.76	0.98	0.460	1.33	0.93	**0.029**	1.01	0.93	**0.015**	1.08	0.97	0.504
Asparagine	2.828–2.862 (d)	0.62	0.97	0.532	1.35	0.92	**0.034**	1.44	0.90	**0.001**	0.55	0.97	0.714
Creatinine	3.036–3.041 (d)	0.97	0.99	0.940	1.11	0.95	**0.047**	0.92	0.95	**0.016**	0.97	0.97	0.550
Formic acid	8.414–8.473 (s)	1.63	0.79	0.059	0.96	0.91	0.461	2.50	0.74	**0.001**	0.72	0.82	0.298
Citric acid	2.502–2.555 (d)	0.93	0.98	0.936	1.28	0.92	0.050	1.18	0.91	**0.011**	0.65	0.97	0.626
Glutamic acid	2.094–2.130 (td)	0.66	0.97	0.411	0.99	0.95	0.185	0.95	0.94	**0.018**	0.84	0.88	0.215
Succinic acid	2.395–2.398 (t)	0.98	0.91	0.745	1.05	0.85	0.361	1.60	0.79	**0.023**	0.65	1.25	0.337
Mannose	4.889–4.904 (d)	0.55	0.97	0.786	1.03	0.93	0.305	1.58	0.86	**0.023**	0.32	0.98	0.677
Tyrosine	6.860–6.917 (dt)	0.70	0.94	0.450	1.11	0.93	0.159	1.09	0.92	**0.024**	0.91	0.89	0.186
3-Hydroxybutyric acid	1.181–1.203 (t)	1.04	0.84	0.879	1.17	0.75	0.523	2.31	0.66	**0.028**	0.94	1.79	0.448
Serine	3.937–3.957 (q)	0.72	0.95	0.222	1.22	0.93	0.104	0.88	0.94	**0.039**	0.92	0.86	0.347
Methanol	3.352–3.360 (s)	0.80	0.91	0.231	0.66	0.96	0.290	0.86	0.94	**0.044**	0.53	0.90	0.619
Isoleucine	0.992–1.017 (d)	0.96	0.99	0.597	0.34	1.02	0.458	0.27	0.97	0.839	1.86	0.69	**0.005**
Isovaleric acid	0.910–0.936 (m)	0.51	0.96	0.580	0.68	0.95	0.324	0.02	1.00	0.859	1.59	0.75	**0.007**
Valine	1.017–1.050 (dd)	0.88	0.99	0.790	0.04	1.00	0.582	0.46	0.97	0.466	1.14	0.82	**0.022**
Leucine	0.947–0.968 (m)	0.78	0.98	0.790	0.22	0.98	0.790	0.43	0.97	0.587	1.09	0.83	**0.028**

* VIP scores were obtained from PLS-DA. † Fold changes were calculated by dividing the value of metabolites in children between different atopic indices. ‡ All FDR-adjusted *p*-values < 0.05, which is in bold, are significant. VIP, Variable Importance in Projection; ppm, parts per million; IgE, immunoglobulin E; FeNO, fractional exhaled nitric oxide; ppb, parts per billion; s, singlet; m, multiplet; d, doublet; t, triplet; td, triplet of doublets; dt doublet of triplets; q, quartet; dd, doublet of doublets.

**Table 3:** Metabolic pathway and function analysis of oxidative stress-related different atopic indices correlated metabolites.

Metabolites	Pathway Name	Total	Hits	Raw *p*	FDR	Function
**8-O** **HdG-related FeNO**						
Threonine	Valine, leucine and isoleucine biosynthesis	8	1	0.020	1.000	Amino acid metabolism
Histidine	Histidine metabolism	16	1	0.040	1.000	Amino acid metabolism
**GPx-related seafood**						
Serine, Threonine	Glycine, serine and threonine metabolism	33	2	0.004	0.254	Amino acid metabolism
Threonine	Valine, leucine and isoleucine biosynthesis	8	1	0.025	0.671	Amino acid metabolism
Serine	D-Amino acid metabolism	15	1	0.047	0.671	Carbohydrate metabolism
**MPO-related mite**						
Serine, Glycine	Glyoxylate and dicarboxylate metabolism	32	2	0.002	0.100	Carbohydrate metabolism
Serine, Glycine	Glycine, serine and threonine metabolism	33	2	0.002	0.100	Amino acid metabolism
Serine	D-Amino acid metabolism	15	1	0.038	0.591	Metabolism of other amino acids
Histidine	Histidine metabolism	16	1	0.040	0.591	Amino acid metabolism
**TAC-related**						
Mannose, Serine	-	-	-	-	-	-

“Total” indicates the total number of compounds included in each pathway; “Hits” represents the number of compounds matched from the uploaded dataset; “Raw *p*” refers to the original *p*-value derived from the enrichment analysis; FDR, false discovery rate; 8-OHdG, 8-hydroxy-2′-deoxyguanosine; FeNO, fractional exhaled nitric oxide; GPx, glutathione peroxidase; MPO, myeloperoxidase; TAC, total anti-oxidant capacity.

## Data Availability

The original contributions presented in this study are included in the article/Supplementary Materials. Further inquiries can be directed to the corresponding author.

## Supplementary Materials

The following supporting information can be downloaded at: https://www.mdpi.com/article/10.3390/antiox15060720/s1, Table S1: PLS-DA parameters and permutation test for distinguishing between different atopic diseases and healthy controls, and with and without atopic indices; Table S2: The VIP score and fold change of metabolites significantly differentially expressed between children with different atopic diseases and healthy controls; Table S3: Metabolic pathway and function analysis of metabolites significantly differentially expressed in children with different atopic indices; Figure S1: Relationships between oxidative stress markers and different atopic indices. The comparisons of (**A**) GPx activity, (**B**) MPO activity, (**C**) 8-OHdG levels, and (**D**) TAC levels were assessed respectively between children with sensitization to seafood, mite, IgE (>100 kU/L), and high FeNO levels (≥20 ppb) and healthy controls; Figure S2: Comparisons of (**A**) total IgE levels, (**B**) *D. pteronyssinus-*, (**C**) *D. farinae-*, (**D**) shrimp-, and (**E**) crab-specific IgE levels, and (**F**) FeNO levels between children with different atopic diseases (Eczema, rhinitis, and asthma) and healthy controls; Figure S3. PLS-DA score plots from the analysis of plasma ^1^H-NMR spectra between (**A**) different atopic diseases and healthy controls, and (**B**) with and without atopic indices.

## Abbreviations

The following abbreviations are used in this manuscript:

ATS	American Thoracic Society
ANOVA	Analysis of variance
BCAA	Branched-chain amino acid
BMI	Body mass index
CGMH	Chang Gung Memorial Hospital
EDTA	Ethylenediaminetetraacetic acid
ELISA	Enzyme-linked immunosorbent assay
ERS	European Respiratory Society
FDR	False discovery rate
FeNO	Fractional exhaled nitric oxide
FRAP	Ferric reducing antioxidant power
glog	Generalized log transformation
GPx	Glutathione peroxidase
GSH	Glutathione
NADPH	Nicotinamide adenine dinucleotide phosphate
NTD	New Taiwan Dollar
NMR	Nuclear magnetic resonance
IgE	Immunoglobulin E
IL	Interleukin
ILC2	Type 2 innate lymphoid cell
iNOS	Inducible nitric oxide synthase
KEGG	Kyoto Encyclopedia of Genes and Genomes database
LC-MS	Liquid chromatography–mass spectrometry
MPO	Myeloperoxidase
NETs	Neutrophil extracellular traps
NO	Nitric oxide
8-OHdG	8-hydroxy-2′-deoxyguanosine
PLS-DA	Partial least squares-discriminant analysis
ppb	parts per billion
ppm	parts per million
ROS	Reactive oxygen species
SCFA	Short-chain fatty acid
SOD	Superoxide dismutase
TAC	Total anti-oxidant capacity
TCA	Tricarboxylic acid
TSP	3-(trimethylsilyl)-propionic-2,2,3,3-d4 acid sodium salt
VIP	Variable Importance in Projection
