# Supplementary material for: Metabolomics-Based Analysis Linking Oxidative Stress-Related Branched-Chain Amino Acid (BCAA) Pathway with Atopic Indices to Childhood Allergies
Source: Antioxidants (Basel). 2026 Jun 5;15(6):720. doi: 10.3390/antiox15060720 (PMC13295394; doi:10.3390/antiox15060720)
Supplement: Supplementary file 1 [file antioxidants-15-00720-s001.zip › antioxidants-4255544-supplementary.pdf]

Supplementary Tables and Figures

Table S1. PLS-DA parameters and permutation test for distinguishing between different atopic diseases and healthy controls, and with and without atopic indices.

	PLS-DA parameters				
	Components*	Q^2	R^2	Q^2/R^2	$P_{permutation}$†
Diseases					
Eczema vs. Controls	1	-0.25	0.16	-1.56	0.608
Rhinitis vs. Controls	1	-0.08	0.06	-1.36	0.666
Asthma vs. Controls	2	-0.41	0.16	-2.60	0.501
Atopic indices					
Seafood sensitization (+/-)	3	-0.10	0.25	-0.42	0.433
Mite sensitization (+/-)	1	-0.01	0.09	-0.11	0.736
IgE (> 100 kU/L) sensitization (+/-)	1	0.05	0.14	0.36	**0.047**
FeNO (≥ 20 ppb) levels (+/-)	2	-0.00	0.26	-0.00	0.992

PLS-DA, partial least squares-discriminant analysis; Q^2, predictive capability; R^2, correlation coefficients. *The number of components based on Q^2 indicates the best classifier of PLS-DA using a 5-fold cross-validation method. †1000 random permutations were performed. IgE, immunoglobulin E; FeNO, fractional exhaled nitric oxide; ppb, parts per billion; All *p*-values < 0.05, which is in bold, are significant.

Table S2. The VIP score and fold change of metabolites significantly differentially expressed between children with different atopic diseases and healthy controls.

Metabolites	Chemical shift, ppm	Eczema vs. Controls			Rhinitis vs. Controls			Asthma vs. Controls		
		VIP score*	Fold change†	*p*‡	VIP score	Fold change	*p*	VIP score	Fold change	*p*
Pyruvic acid	2.358- 2.372(s)	2.71	1.27	**0.024**	0.69	0.96	0.454	1.35	1.15	0.114
Creatinine	3.036- 3.041(d)	0.31	1.01	0.716	1.39	0.95	**0.035**	0.65	1.04	0.383
Methionine	2.610- 2.653(t)	0.44	1.02	0.708	1.80	1.07	**0.044**	0.91	1.10	0.310
Asparagine	2.828- 2.862(d)	1.91	0.86	0.058	0.01	1.00	0.995	0.80	0.98	0.876
Alanine	1.455- 1.490(q)	1.89	1.14	0.111	0.52	0.97	0.581	1.24	1.12	0.250
3-Hydroxybutyric acid	1.181- 1.203(t)	1.78	0.52	0.468	2.43	0.69	0.213	1.43	0.47	0.401
Isoleucine	0.992- 1.017(d)	1.52	0.84	0.226	0.75	0.94	0.444	0.93	1.04	0.627
Ribose	4.924- 4.947(d)	1.45	0.87	0.268	1.07	0.94	0.311	2.80	0.82	0.085
Succinic acid	2.395- 2.398(t)	1.42	0.75	0.408	1.96	0.82	0.141	0.77	0.76	0.508
L-Acetylcarnitine	3.182- 3.187(s)	1.41	1.09	0.156	1.01	0.95	0.184	1.02	1.08	0.170
Glycerol	3.635- 3.650 (dd)	1.34	1.09	0.204	1.15	0.94	0.157	0.74	1.01	0.700
Methanol	3.352- 3.360(s)	1.29	0.87	0.260	0.60	0.98	0.541	0.76	1.08	0.343
Tyrosine	6.860-6.917(dt)	1.16	1.10	0.303	0.15	0.98	0.865	1.05	1.04	0.521
Ornithine	3.041- 3.075(t)	1.14	1.07	0.275	0.66	0.96	0.397	0.81	1.07	0.303
Proline	3.309- 3.339(m)	1.12	1.08	0.327	0.27	1.00	0.767	1.41	1.18	0.090
Acetone	2.212- 2.234(s)	1.00	1.06	0.575	1.67	0.83	0.223	0.34	0.96	0.781
Formic acid	8.414- 8.473(s)	0.49	0.95	0.773	2.06	0.87	0.145	0.77	1.12	0.529

Acetoacetic acid	2.272- 2.278(s)	0.99	0.82	0.538	1.58	0.87	0.214	0.42	0.87	0.838
Mannose	4.889- 4.904(d)	0.43	0.92	0.791	1.52	1.07	0.220	0.68	0.91	0.807
Lactic acid	1.304- 1.340(t)	0.81	1.06	0.539	1.40	0.93	0.192	0.86	1.12	0.429
Glycine	3.548- 3.562(s)	0.24	1.01	0.808	1.13	0.95	0.121	0.73	1.06	0.281
Isovaleric acid	0.910- 0.936(m)	0.60	0.93	0.625	0.16	0.98	0.871	1.38	0.95	0.670
Serine	3.937- 3.957(q)	0.47	0.96	0.634	0.79	0.96	0.289	1.24	1.02	0.799
Creatine	3.915- 3.928(s)	0.07	0.99	0.946	0.33	0.97	0.682	1.15	1.02	0.789
Arabinose	4.509- 4.514(d)	0.14	1.02	0.922	0.82	0.97	0.504	1.11	0.97	0.773
Sarcosine	2.704- 2.717(s)	0.15	0.92	0.928	0.04	0.97	0.977	1.07	1.16	0.561

*VIP scores were obtained from PLS-DA. †Fold changes were calculated by dividing the value of metabolites in children with atopic diseases and healthy controls. ‡All FDR-adjusted *p*-values < 0.05, which is in bold, are significant. VIP, Variable Importance in Projection; ppm, parts per million; s, singlet; d, doublet; t, triplet; q, quartet; dd, doublet of doublets; dt, doublet of triplets; m, multiplet.

Table S3. Metabolic pathway and function analysis of metabolites significantly differentially expressed in children with different atopic indices.

Metabolites	Pathway Name	Total	Hits	Raw *p*	FDR	Function
**Seafood (+/-)**						
Threonine	Valine, leucine and isoleucine biosynthesis	8	1	0.015	0.607	Amino acid metabolism
Glucose	Starch and sucrose metabolism	18	1	0.034	0.616	Carbohydrate metabolism
Acetic acid	Pyruvate metabolism	23	1	0.043	0.616	Carbohydrate metabolism
Acetic acid	Glycolysis / Gluconeogenesis	26	1	0.049	0.616	Carbohydrate metabolism
**Mite (+/-)**						
Histidine	Histidine metabolism	16	1	0.040	0.650	Amino acid metabolism
**Total IgE > 100 kU/L (+/-)**						
Serine, Formic acid, Citric acid, Glycine	Glyoxylate and dicarboxylate metabolism	32	4	<0.001	0.005	Carbohydrate metabolism
Asparagine, Citric acid, Succinic acid	Alanine, aspartate and glutamate metabolism	28	3	<0.001	0.004	Amino acid metabolism
Threonine, Serine, Glycine	Glycine, serine and threonine metabolism	33	3	0.002	0.043	Amino acid metabolism
Succinic acid, 3-Hydroxybutyric acid	Butanoate metabolism	15	2	0.005	0.106	Carbohydrate metabolism
Succinic acid, Citric acid	Citrate cycle (TCA cycle)	20	2	0.009	0.150	Carbohydrate metabolism
Glucose, Mannose	Galactose metabolism	27	2	0.017	0.192	Carbohydrate metabolism
Tyrosine	Phenylalanine, tyrosine and tryptophan biosynthesis	4	1	0.030	0.302	Amino acid metabolism
**FeNO ≥ 20 ppb (+/-)**						

Isoleucine, Valine, Leucine	Valine, leucine and isoleucine biosynthesis	8	3	<0.001	<0.001	Amino acid metabolism
Isoleucine, Valine, Leucine	Valine, leucine and isoleucine degradation	40	3	<0.001	0.002	Amino acid metabolism
Valine	Pantothenate and CoA biosynthesis	20	1	0.050	1.000	Metabolism of cofactors and vitamins

Total is the total number of compounds in the pathway; the Hits is the actually matched number from the user uploaded data; the Raw *p* is the original *p*-value calculated from the enrichment analysis; FDR, false discovery rate, the portion of false positives above the user-specified score threshold; IgE, immunoglobulin E; FeNO, fractional exhaled nitric oxide; ppb, parts per billion.

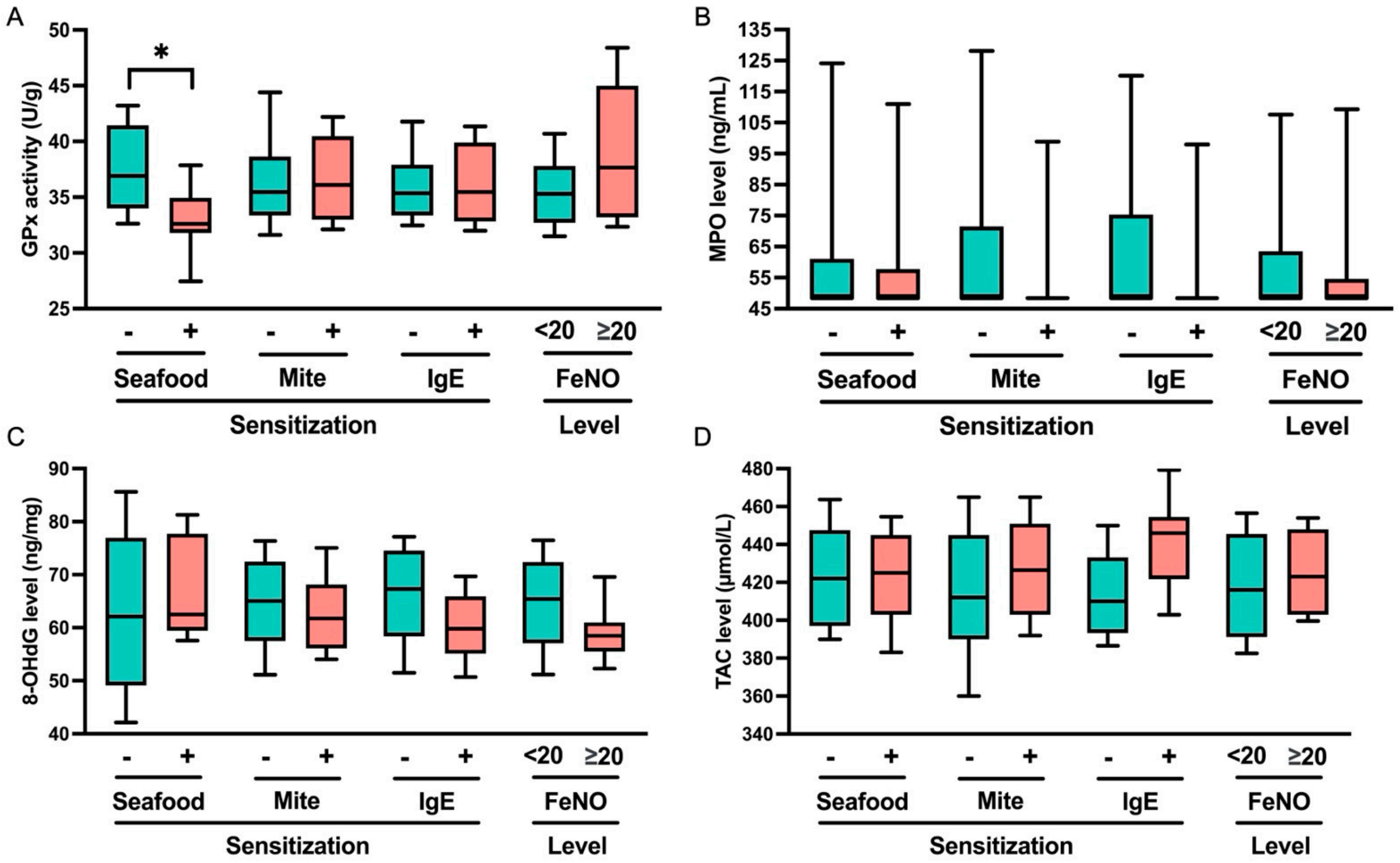

Figure S1. Relationships between oxidative stress markers and different atopic indices. The comparisons of GPx activity (A), MPO activity (B), 8-OHdG levels (C), and TAC levels (D) were assessed respectively between children with sensitization to seafood, mite, IgE (> 100 kU/L), and high FeNO levels (≥ 20 ppb) and healthy controls. GPx, glutathione peroxidase; MPO, myeloperoxidase; 8-OHdG, 8-hydroxy-2'-deoxyguanosine; TAC, total anti-oxidant capacity; IgE, immunoglobulin E; FeNO, fractional exhaled nitric oxide; ppb, parts per billion. *p-value < 0.05.

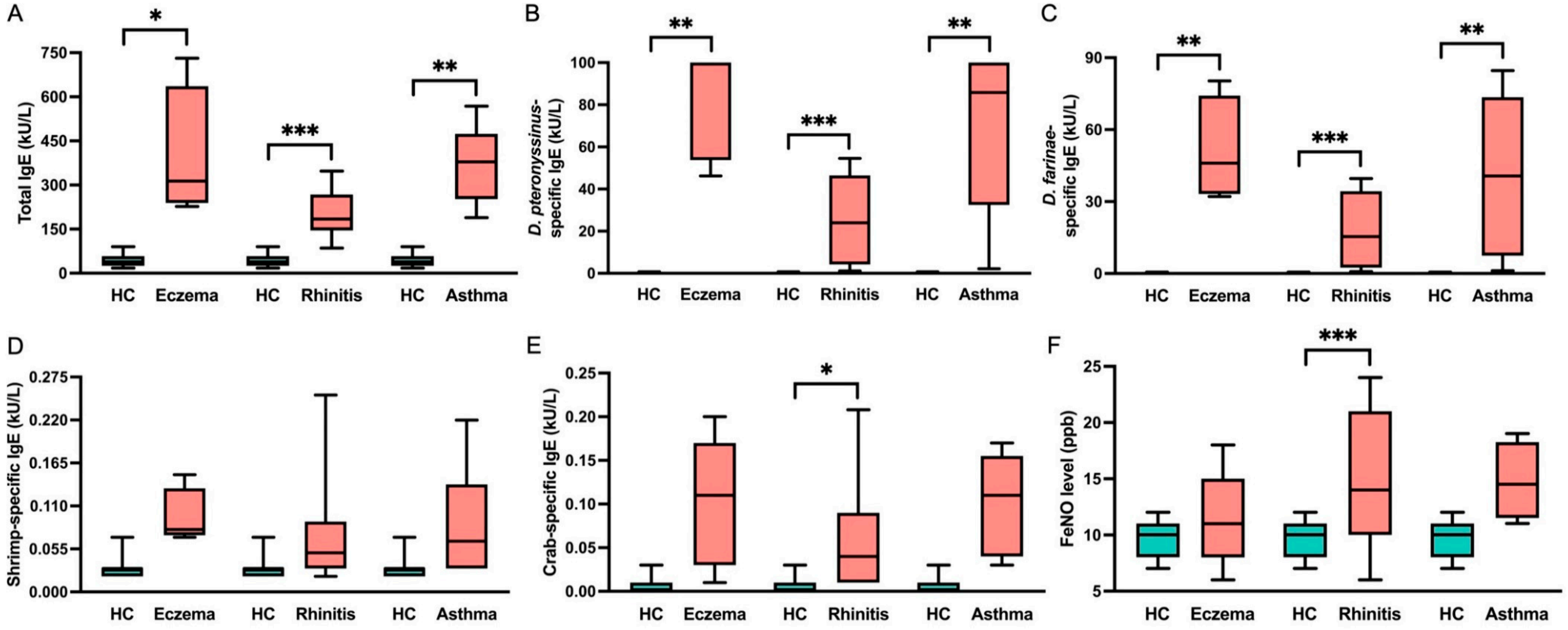

Figure S2. Comparisons of total IgE levels (A), *D. pteronyssinus*- (B), *D. farinae*- (C), shrimp- (D), and crab- (E) specific IgE levels, and FeNO levels (F) between children with different atopic diseases (Eczema, rhinitis, and asthma) and healthy controls. HC, healthy controls. * p-value < 0.05; **p-value < 0.01; ***p-value < 0.001.

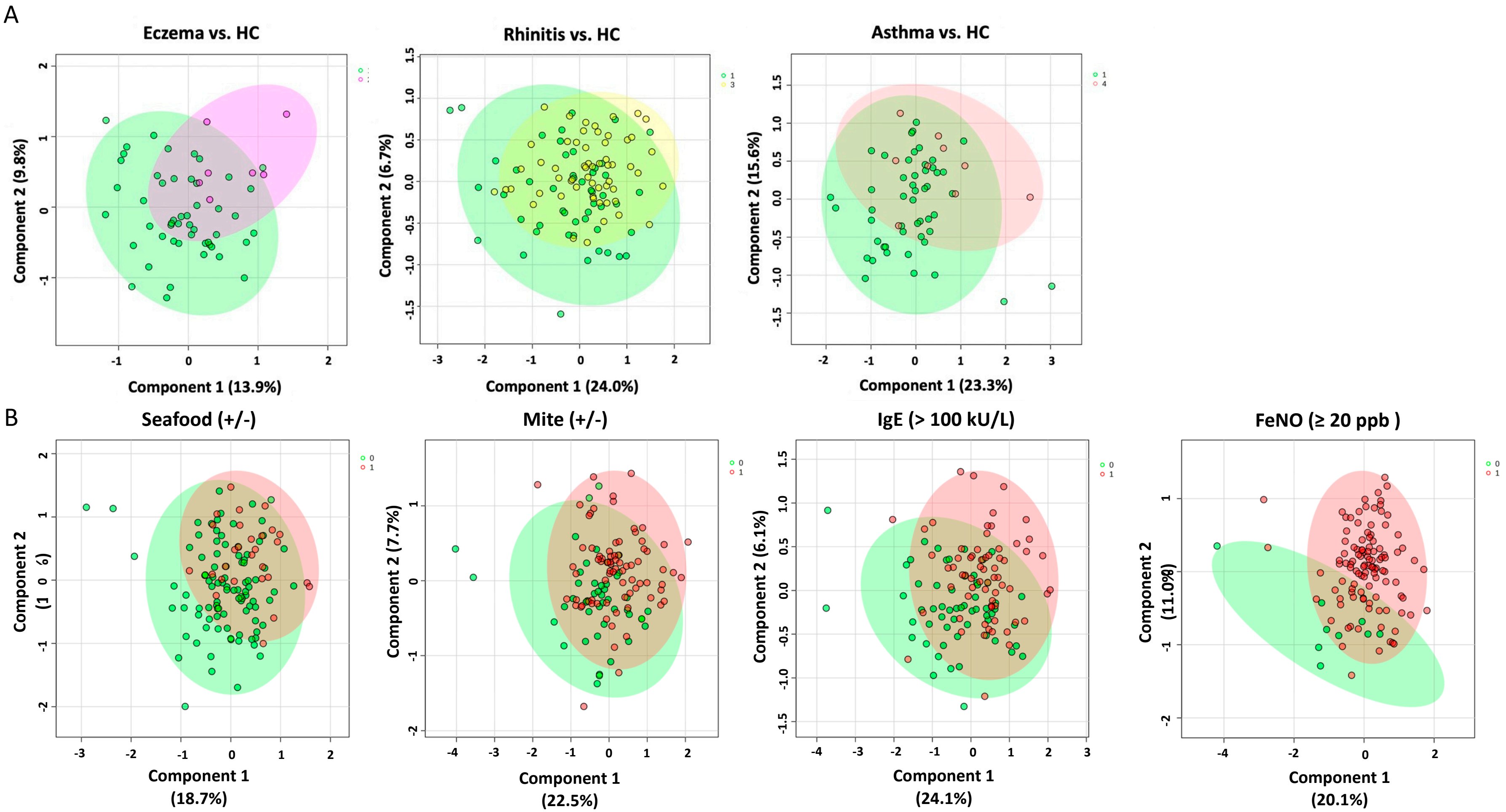

Figure S3. PLS-DA score plots from the analysis of plasma ^{1}H-NMR spectra between different atopic diseases and healthy controls (A), and with and without atopic indices (B). x axis, component 1 (% of total variance); y axis, component 2 (% of total variance). HC, healthy controls; IgE, immunoglobulin E; FeNO, fractional exhaled nitric oxide; ppb, parts per billion.
